# Influence of bone densitometry on the anti-osteoporosis treatment after fragility hip fracture

**DOI:** 10.1007/s40520-018-1094-7

**Published:** 2018-12-17

**Authors:** Peiwen Wang, Yizhong Li, Huafeng Zhuang, Haiming Yu, Siqing Cai, Hao Xu, Zhenhui Chen, Jinkuang Lin, Xuedong Yao

**Affiliations:** 1 The Department of Orthopaedics, The Second Affiliated Hospital of Fujian Medical University, Zhongshan North Road 34, Quanzhou, 362000 Fujian China; 2 The Department of Radiology, The Second Affiliated Hospital of Fujian Medical University, Quanzhou, Fujian China

**Keywords:** Hip fracture, Bone mineral density assessment, Osteoporosis, Treatment

## Abstract

**Background:**

Fragility fracture significantly increases risk of future fracture. The fragility fracture cycle should be disrupted. The secondary fracture prevention is important for the patients with fragility hip fracture. The pharmacotherapy for osteoporosis is important for prevention of new fracture. However, many patients with hip fracture do not receive osteoporosis treatment. This retrospective study investigates the influence of bone mineral density (BMD) assessment on the initiation of anti-osteoporosis medications in the hospitalized patients with fragility hip fracture.

**Methods:**

This retrospective research enrolled 1211 patients with fragility hip fracture 50 years of age and older. Among 1211 patients aged from 50 to 103 years with the average age of 77.83 ± 9.95 years, there were 807 females and 404 males. There were 634 fractures of femoral neck and 577 intertrochanteric fractures of femur. We examined whether patients had received bone mineral density assessment and received anti-osteoporosis therapy during the period of hospitalization. The patients were divided into BMD assessment group and no BMD assessment group. Measurement data were expressed as mean ± standard deviation and compared with *t* test. All parameters of groups were compared with Chi-square test.

**Results:**

Of 1211 patients, 331 (27.33%) had received BMD assessment and 925 (76.38%) had received anti-osteoporosis drugs during the period of hospitalization. The rate of bisphosphonate use was lower and only 11.31% in the total patients. The anti-osteoporosis treatment rate was 93.66% in the patients receiving BMD assessment and 69.89% in the patients without BMD assessment (*p* < 0.01). The zoledronate use significantly increased from 6.7% in the patients without BMD assessment to 23.56% in the patients receiving BMD assessment (*p* < 0.01).

**Conclusions:**

BMD assessment is a good basis for communication between patients and orthopedic surgeons. BMD assessment significantly increases the initiation of osteoporosis treatment and bisphosphonate use in the patients with hip fracture during the period of hospitalization.

## Background

Fragility hip fracture is common in the postmenopausal women and old individuals, and is a leading cause of death in older adults. The patients with fragility hip fracture in the world were 1,500,000 in 2000 and were estimated to be 6,260,000 in 2050 [1, 2]. The incidence of hip fracture over the age of 50 in China was 229/1,000,000 in the female and 129/1,000,000 in the male, and ascended at a rate of about 10% each year in 2002–2006 [3]. The first-year mortality after hip fracture was 16–30% [1, 2, 4]. The fragility hip fracture is highly associated with osteoporosis which is characterized by low bone mineral density and bone strength. The majority of patients with hip fractures show low bone density and hip structure deterioration under bone mineral density (BMD) assessment and hip structure analysis [5]. BMD also is an indicator for the diagnosis of osteoporosis, prediction of fracture risk, initiation of osteoporosis treatment, and evaluation of effect of drugs. The anti-osteoporosis medications were recommended for patients with BMD *T* score ≤ − 2.5 or with fragility hip fracture by European guidance for the diagnosis and management of osteoporosis in postmenopausal women and UK clinical guideline for the prevention and treatment of osteoporosis [6, 7]. However, the reality is that many patients with hip fracture do not receive osteoporosis treatment. The treatment gap of osteoporosis is evident around the world. Although many factors influence the treatment of osteoporosis, patient understanding of the results of BMD assessment is critical important for the improvement of osteoporosis treatment [8]. In this retrospective study, we investigate the influence of BMD assessment on the initiation of anti-osteoporosis medications in the hospitalized patients with fragility hip fracture.

## Methods

### Study population

This retrospective research enrolled 1211 patients aged ≥ 50 years who sustained hip fragility fractures. The patients were admitted to the Second Affiliated Hospital of Fujian Medical University in China from January 2010 to December 2015. Patients were not included if they suffered from pathological fractures caused by malignant tumors, and high-energy fracture and re-fracture of same hip. The patients’ medical data were retrospectively reviewed. Among 1211 patients aged from 50 to 103 years with the average age of 77.83 ± 9.95 years. There were 807 females and 404 males. 634 patients had fractures of femoral neck and 577 patients had intertrochanteric fractures of femur. 1069 patients received the surgical treatment and 142 had conservative treatment. Before this hip fracture, 61 patients had the previous non-traumatic fractures including 42 patients with contralateral hip fractures, 6 with vertebral fractures, 4 with proximal humeral fractures, and 7 with distal radius fractures. There were 787 (65%) patients combined with internal diseases and 408 (34%) with more than or equal to two types of internal disease. The main combined diseases included hypertension, coronary atherosclerotic heart disease, stroke sequela, lacunar infarction, Parkinson’s syndrome, senile dementia, diabetes mellitus, hyperthyroidism, chronic bronchitis, asthma, chronic obstructive pulmonary disease, gastric /duodenal ulcer, liver cirrhosis, rheumatoid arthritis and urinary calculi, and renal insufficiency. Charlson comorbidity index (CCI) was 0–6. Anti-osteoporosis therapy was carried out in eight patients before the hip fracture. The patients were divided into BMD assessment group and no BMD assessment group according to BMD assessment. Of 1211 patients, 331 had the BMD assessment with DXA and 880 had not the BMD assessment during the period of hospitalization. To study the effect of BMD assessment on the initiation of anti-osteoporosis medications in the hospitalized patients with hip fragility fracture, we identified the patients receiving anti-osteoporosis therapy in the patients with BMD assessment and without BMD assessment, and the osteoporosis treatments were divided into no anti-osteoporosis medication, supplementation of calcium and vitamin D, non-bisphosphonate medication (calcium + vitamin D + non-bisphosphonate), and bisphosphonate medication (calcium + vitamin D + bisphosphonate).

## Statistical analysis

Baseline characteristics of the patient were summarized. Data analyses were performed using SPSS statistical software (SPSS, version 19.0; SPSS Inc, Chicago, Illinois). Measurement data were expressed as mean ± standard deviation and compared with *t* test. All parameters of groups were compared with Chi-square test. *p* < 0.05 was considered as statistically significant.

## Results

BMD measurement: 331 of 1211 (27.33%) patients had the BMD assessment. 316 patients had BMD *T* score ≤ − 2.5 at femoral neck or lumbar spine, and 15 cases had BMD *T* score between − 1 and − 2.5 at femoral neck and/or lumbar spine in BMD assessment.

Anti-osteoporosis treatment: 925 of 1211 (76.38%) patients had anti-osteoporosis medications during the period of hospitalization. Of these 925 patients, 100 patients had the supplementation of calcium and vitamin D, 588 patients had calcium + vitamin D + non-bisphosphonate medication, and 137 patients had calcium + vitamin D + bisphosphonate. Calcitonin, calcitriol, and alfacalcidol were often used to the patients in the calcium + vitamin D + non-bisphosphonate medication group and zoledronate was only one of the bisphosphonates used in our patients during the period of hospitalization (Table 1).

**Table 1:** Baseline characteristics of hip fracture patients in BMD assessment group and no BMD assessment group

Characteristic	BMD assessment group	No BMD assessment group	*χ* ^2^	*p*
Patients number	331	880		
Age, mean (SD)	78.21 ± 9.65	77.69 ± 9.84		> 0.05
50–64, *n* (%)	36 (10.88%)	118 (13.41%)	1.39032	> 0.05
65–79, *n* (%)	119 (35.95%)	330 (37.50%)	0.24716	> 0.05
≥ 80, *n* (%)	176 (53.17%)	432 (49.09%)	1.60262	> 0.05
Male	109 (32.93%)	295 (33.52%)	0.03795	> 0.05
Female	222 (67.07%)	585 (66.48%)	0.03795	> 0.05
Combined internal disease, *n* (%)	199 (60.12%)	588 (66.82%)	4.74153	< 0.05
< 2 Combined internal disease, *n* (%)	115 (34.74%)	264 (30.00%)	2.51672	> 0.05
≥ 2 Combined internal disease, n (%)	84 (25.37%)	324 (36.82%)	14.092	< 0.01
Fracture of femoral neck *n* (%)	187 (56.50%)	447 (50.80%)	3.13286	> 0.05
Intertrochanteric fracture *n* (%)	144 (43.50%)	433 (49.20%)	3.13286	> 0.05

Effect of BMD assessment on the initiation of anti-osteoporosis medications: the anti-osteoporosis rate was 93.66% in the patients receiving BMD assessment and 69.89% in the patients without BMD assessment (*p* < 0.01). The zoledronate use significantly increased from 6.7% in the patients without BMD assessment to 23.56% in the patients receiving BMD assessment (*p* < 0.01) (Table 2).

**Table 2:** Comparison of anti-osteoporosis medications between BMD assessment group and no BMD assessment group

Characteristic	BMD assessment group	No BMD assessment group	*χ* ^2^	*p*
Patients’ number	331	880		
Anti-osteoporosis group, *n* (%)	310 (93.66%)	615 (69.89%)	75.3316	< 0.01
Calcium + vitamin D, *n* (%)	38 (11.48%)	62 (7.05%)	6.24468	< 0.05
Non-bisphosphonate medication, *n* (%)	194 (58.61%)	494 (56.14)	0.59997	> 0.05
Bisphosphonate medication, *n* (%)	78 (23.56%)	59 (6.70%)	68.1501	< 0.01

## Discussion

Fragility fracture significantly increases the risk of future fracture. The fragility fracture cycle should be disrupted. The secondary fracture prevention is important for the patients with fragility fracture including hip fracture and vertebral fractures. In a retrospective, observational cohort study including a total of 103,852 women ≥ 65 years of age with a fragility fracture, 8.3% of women had a subsequent fracture within 1 year post-fracture and only 27.7% of patients initiated treatment within 12 months of fracture, although the patients receiving osteoporosis treatment had a significantly lower risk of fractures compared to those without osteoporosis treatment (6.4 vs 9.0%, *p* < 0.001) [9]. Wilk et al. conducted a retrospective study including a total of 47,171 women aged ≥ 50 years with fragility fracture in 2014 and only 18% patients received osteoporosis therapy within 90 days and 23% within 1 year post-fracture in USA [10]. Klop C et al. studied the trends and determinants of anti-osteoporosis drug prescribing in 30,516 patients aged 50 years or older after hip fracture in the UK, anti-osteoporosis drugs after hip fracture increased from 7% in 2000 to 46% in 2010, and 94% of 6684 patients receiving anti-osteoporosis therapy took bisphosphonates [11]. The treatment rate for osteoporosis in our study was 76.38% during the period of hospitalization for the management of hip fracture and better than those reported above. However, only 11.31% patients received the bisphosphonate (zoledronate) medication. Bisphosphonates are the first-line treatments recommended by the guidelines [6, 7]. Low treatment rate and bisphosphonate use suggested that the orthopedic surgeons and physicians did not adhere to the guidelines for osteoporosis.

The treatment gap of osteoporosis is evident throughout the world. No treatment is often in the patients diagnosed with osteoporosis. In a retrospective study including a total of 65,344 patients, 42,033 patients (64.3%) received no medication and 23,311 patients (35.7%) received osteoporosis medication, of which 20,200 patients received bisphosphonates and 3111 patients received non-bisphosphonate medication [12]. What are reasons for no treatment of osteoporosis? Lindsay BR et al. investigated reasons for non-treatment of osteoporosis from the osteoporosis patient’s perspective and found that barriers to initiation of osteoporosis treatment included the use of alternative treatments such as vitamins/supplements and fear of side effects [13]. Yu et al. found that, among US osteoporotic women aged 55 and older, the primary reason for no initiation of osteoporosis therapy was concern over side effects [14]. The excessive worry for drug side effect is unnecessary. Several studies confirmed that bisphosphonates could significantly reduce the subsequent fractures and had good safety for long use [15]. The importance of osteoporosis therapy and drug safety needs orthopedic surgeons to communicate with patients. However, in a survey of 2910 orthopedic surgeons, the majority of orthopedic surgeons questioned lacked knowledge and sufficient training in the management of osteoporosis [16]. Obviously, the education of osteoporosis knowledge is necessary for both orthopedic surgeons and patients. The treatment can be improved if orthopedic surgeons and patients have a full understanding to the harm of osteoporosis.

The diagnosis of osteoporosis is important in patients receiving pharmacological therapy after hip fracture. The studies showed that initiation of drug therapy is significantly linked to patient understanding of DXA results [8, 17] and that physicians also relied heavily on BMD *T* score to decide on the initiation of osteoporosis treatment [18]. In Asian countries, a retrospective study confirmed that osteoporosis diagnosis and treatment was driven by BMD measurement [19]. BMD measured by dual-energy X-ray absorptiometry (DXA) is “gold standard “of diagnosing osteoporosis. DXA results help the patients to know the status of bone health and fracture risk, and help physicians and orthopedic surgeons to evaluate the fracture risk, to make decision of initiating the medication for osteoporosis, and to evaluate the effect of drugs. Bone densitometry result is a good basis for the communication between patients and orthopedic surgeons. Patients’ understanding of BMD results is an important component in the initiation of medication after BMD assessment. A study confirmed that all fractured patients with osteoporosis or osteopenia received bisphosphonate medications after diagnosis with densitometry [20]. At the same time, orthopedic surgeons’ decision is crucial. Orthopedic surgeons are often the first clinicians to manage the fragility fracture of osteoporosis consequences and have objective evidence of patient’s bone health. Orthopedic surgeons have a unique position in the management of osteoporosis. Studies in Canada have shown that, if the surgeons order the BMD assessment, the patient is likely to get treatment for osteoporosis and patient compliance goes up [21]. A prospective cohort study confirmed that orthopedic surgeon’s awareness can improve osteoporosis treatment following hip fracture. The BMD assessment and treatment rate after hip fracture increased twofold after orthopedic surgeons’ involvement in the treatment of osteoporosis [22]. In our study, orthopedic surgeons did not only depend on the results of BMD to decide the initiation of the osteoporosis medication, because BMD assessment rate was significantly lower than treatment rate of osteoporosis. Patients’ age, comorbidities, number of medications, and previous fractures might influence the decision of the orthopedic surgeon regarding the need of initiation therapy. However, BMD assessment significantly increased the rates of osteoporosis treatment from 69.89 to 93.66%, and increased bisphosphonate use from 6.7 to 23.56% compared with no BMD assessment in the patients with hip fracture. We think that the baseline BMD assessment is necessary and good for the patients to receive the first-line treatment for osteoporosis. BMD is an important indicator for patients and orthopedic surgeons to know the effect of drugs and helpful for improvement of patients’ compliance of drug. Although osteoporosis involves multiple disciplines, orthopedic surgeons may play an active part in care of patients with osteoporotic fractures and initiate the diagnosis and treatment of osteoporosis for patients with fragility hip fracture as soon as possible.

In conclusion, BMD assessment is a good tool for understanding of bone health and a good basis for communication between patients and orthopedic surgeons. BMD assessment significantly increases the initiation of osteoporosis treatment in the patients with hip fracture during the period of hospitalization.

## Abbreviations

BMD: Bone mineral density
DXA: Dual-energy X-ray absorptiometry
